# Sparse kernel models provide optimization of training set design for genomic prediction in multiyear wheat breeding data

**DOI:** 10.1002/tpg2.20254

**Published:** 2022-08-31

**Authors:** Marco Lopez‐Cruz, Susanne Dreisigacker, Leonardo Crespo‐Herrera, Alison R Bentley, Ravi Singh, Jesse Poland, Sandesh Shrestha, Julio Huerta‐Espino, Velu Govindan, Philomin Juliana, Suchismita Mondal, Paulino Pérez‐Rodríguez, Jose Crossa

**Affiliations:** ^1^ Dep. of Epidemiology and Biostatistics Michigan State Univ. East Lansing MI USA; ^2^ Global Wheat Program International Maize and Wheat Improvement Center (CIMMYT) Texcoco Mexico; ^3^ Dep. of Agronomy Kansas State Univ. Manhattan KS USA; ^4^ Campo Experimental Valle de Mexico, Instituto Nacional de Investigaciones Forestales Agricolas y Pecuarias (INIFAP) Chapingo Mexico; ^5^ Colegio de Postgraduados Montecillos Mexico

## Abstract

The success of genomic selection (GS) in breeding schemes relies on its ability to provide accurate predictions of unobserved lines at early stages. Multigeneration data provides opportunities to increase the training data size and thus, the likelihood of extracting useful information from ancestors to improve prediction accuracy. The genomic best linear unbiased predictions (GBLUPs) are performed by borrowing information through kinship relationships between individuals. Multigeneration data usually becomes heterogeneous with complex family relationship patterns that are increasingly entangled with each generation. Under these conditions, historical data may not be optimal for model training as the accuracy could be compromised. The sparse selection index (SSI) is a method for training set (TRN) optimization, in which training individuals provide predictions to some but not all predicted subjects. We added an additional trimming process to the original SSI (trimmed SSI) to remove less important training individuals for prediction. Using a large multigeneration (8 yr) wheat (*Triticum aestivum* L.) grain yield dataset (n = 68,836), we found increases in accuracy as more years are included in the TRN, with improvements of ∼0.05 in the GBLUP accuracy when using 5 yr of historical data relative to when using only 1 yr. The SSI method showed a small gain over the GBLUP accuracy but with an important reduction on the TRN size. These reduced TRNs were formed with a similar number of subjects from each training generation. Our results suggest that the SSI provides a more stable ranking of genotypes than the GBLUP as the TRN becomes larger.

## Core Ideas

- Training set optimization is desirable when using large heterogeneous, multigeneration data.
- The SSI and TSSI provide customized TRNs for each selection candidate.
- TSSI provides a reduced TRN that maximizes prediction accuracy and minimizes MSE.
- All generations are still present in the reduced TRN with an equal number of individuals from each generation.

**Abbreviations**
GBLUP: genomic best linear unbiased prediction
GBS: genotyping‐by‐sequencing
GS: genomic selection
LD: linkage disequilibrium
MSE: mean squared error
SSI: sparse selection index
TRN: training set
TSSI: trimmed sparse selection index
TST: prediction (testing) set

## 1 INTRODUCTION

Research in plant breeding methods is a crucial first step to accelerate breeding progress to increase food production and subsequently improve food security on a global scale. Genomic selection (GS; Meuwissen et al., 2001) is being implemented in many plant breeding programs to accelerate the process of the development of new crop cultivars (Crossa et al., 2017). Genomic selection consists of genotyping and phenotyping individuals in a reference population (training set [TRN]) and, with the help of calibration methods (e.g., linear models or machine learning tools), predicting the breeding values of the unobserved phenotypes of the candidates for selection that were only genotyped (prediction set [TST]). Genomic selection shortens the breeding cycle because data‐driven predictions of unphenotyped individuals support a more comprehensive and reliable selection of candidate individuals in earlier breeding generations than is possible via traditional means.

There are several advantages of GS over conventional selection including reducing costs by saving resources required for labor‐intensive phenotyping, saving time needed for variety development by reducing the cycle length, increasing the selection intensity and thus capturing greater gain per unit time, selecting traits that are very difficult to measure, and offering opportunities to improve the accuracy of the selection process. Undoubtedly, a successful implementation of GS strongly depends on many factors such as the heritability of the trait, family relationships, marker quality and density, linkage disequilibrium (LD), genotype × environment interaction, and composition of the TRNs and TSTs (Crossa et al., 2017). However, the quality of the training data (that was phenotyped and genotyped) for predicting the breeding values of the individuals in the TST (that was only genotyped) is one of the most influential factors for increasing genomic prediction accuracy (Lopez‐Cruz & de los Campos, 2021).

A variety of models and methods can be described for genomic prediction using information on phenotypes and molecular markers. The most commonly used model is the linear additive genomic best linear unbiased predictor (GBLUP; VanRaden, 2007, 2008) that uses an additive genomic relationship matrix derived from markers (VanRaden, 2008) for predictions. Some genetic complexities (e.g., gene × gene epistatic interactions) can be addressed by using semiparametric genomic regression to account for nonadditive variation. One semiparametric genomic regression is the reproducing kernel Hilbert spaces method with the Gaussian kernel (de los Campos et al., 2009; Gianola et al., 2006, 2011, 2014; Morota & Gianola, 2014; Morota et al., 2013) that models nonlinear relationships between phenotype and genotype (Crossa et al., 2019).

Genomic prediction in wheat breeding plays a fundamental role, as it has the potential to increase the rate of genetic gain relative to traditional phenotypic and pedigree‐based selection. Despite the documented benefits of applying GS in plant breeding and the various models and methods available for assessing prediction accuracy, several limiting factors exist that impede its full implementation: (a) cost of genotyping, (b) insufficient number of individuals in the TRN, (c) training individuals that do not represent or do not offer any increase on the prediction accuracy of the individuals in the TST, and (d) high heterogeneity between training and predicted individuals. Therefore, it is particularly important to identify an optimal TRN for individuals in the TST. Usually, the algorithm employed for selecting an optimal TRN maximizes the relationship with the individuals in the TST whilst minimizing the correlation among the training data. The algorithm then makes the prediction and finally selects the candidates with the highest genomic estimated breeding values (Rincent et al., 2012; Akdemir et al., 2015; Pszczola & Calus, 2016; Akdemir & Isidro‐Sanchez, 2019).

Most of the methods for TRN optimization assume that a single TRN is optimal for all the individuals in the TST. However, this is a weak assumption that is rarely proven because some lines in the TRN can increase prediction for some, but not all, lines in the TST. Results from different studies (Lorenz & Smith, 2015; Lopez‐Cruz & de los Campos, 2021; Lopez‐Cruz et al., 2021) indicate that borrowing information from training individuals distantly related to the individuals in the TST might have a negative impact on the prediction accuracy because of the heterogeneity of allele frequency and LD between TRN and TST. That is, the prediction of individuals in the TST relies on the ability to borrow similar alleles and haplotypes from the training data. Evidence suggests that when training individuals are distantly related to those in the TST, the genomic prediction accuracy can even be reduced (de los Campos et al., 2013).

To overcome the main problem of inclusion in the TRN of individuals distantly related to those to be predicted (with the known detrimental consequences on prediction accuracy), Lopez‐Cruz and de los Campos (2021) developed a genomic prediction method that efficiently optimizes the TRN by finding subsets of individuals for each individual in the TST. The authors proposed integrating sparsity into a selection index (sparse selection index [SSI]) by means of a regularization parameter (λ ≥ 0), noting that the SSI can be seen as a sparse version of the GBLUP. Results of applications of the SSI showed increased genomic prediction accuracy for grain yield between 5 and 10% in wheat (*Triticum aestivum* L.) (Lopez‐Cruz & de los Campos, 2021) and between 5 and 17% in maize (*Zea mays* L.) (Lopez‐Cruz et al., 2021) relative to the GBLUP.

Several analyses based on different original models and methods have been performed on the extensive historical data set generated by the Global Wheat Program of the International Maize and Wheat Improvement Center (CIMMYT). Pérez‐Rodríguez et al. (2017) analyzed a CIMMYT wheat data set to evaluate the prediction performance of phenotypes across environments using single‐step genomic and pedigree models incorporating genotype × environment interactions. The data set comprised a total of 58,798 lines derived from years 2009 through 2016 evaluated at several environments in Mexico and southern Asia with the objective of breeding for high grain yield and resilience to drought, heat, and late heat (Crespo‐Herrera et al., 2021). Lopez‐Cruz and de los Campos (2021) presented the novel SSI optimization method using a subset of this CIMMYT data containing 29,484 wheat lines corresponding to the environments in Mexico only. Howard et al. (2019) used the multiple‐environment CIMMYT wheat data from years 2014 through 2017 for 35,403 1‐yr tested lines to evaluate the prediction accuracy of models including pedigree and genomic information. Pérez‐Rodríguez et al. (2020) analyzed a larger CIMMYT data set including 45,099 wheat lines derived from years 2014 through 2018 for a single environment to evaluate the predictive power of historical data. More recently, Dreisigacker et al. (2021) presented updated pedigree and genomic prediction analyses using an extended data set including years 2014 through 2019 for a total of 52,242 wheat genotypes.

Based on the above considerations and on the previous analyses performed on part of the extensive CIMMYT wheat multigeneration data, the present study used all historical data since 2014 to 2021 with the main objectives of (a) computing the genomic prediction accuracy of the SSI and the GBLUP and (b) to examine if adding more previous years to the TRN increases the genome‐enabled accuracy of prediction of grain yield performance of the wheat lines in 2019, 2020, and 2021 cycles. An important motivation of this research was to use the SSI to examine the effective number of wheat lines in the TRN that participate on the prediction of the wheat lines in the TST. Furthermore, we added an extra trimming process to the original SSI method to further remove less important training individuals for prediction. This is named trimmed SSI (TSSI) to distinguish it from the original SSI application. We used two metrics to assess the genomic prediction accuracy: (a) the correlation between the observed and predicted values and (b) the mean squared error (MSE) of prediction. The dataset used in this study includes a total of 68,836 wheat lines genotyped with 11,293 genotyping‐by‐sequencing (GBS) markers that were evaluated during eight cycles (2014–2021).

**Core Ideas**

- Training set optimization is desirable when using large heterogeneous, multigeneration data.
- The SSI and TSSI provide customized TRNs for each selection candidate.
- TSSI provides a reduced TRN that maximizes prediction accuracy and minimizes MSE.
- All generations are still present in the reduced TRN with an equal number of individuals from each generation.

## 2 MATERIALS AND METHODS

### 2.1 Phenotypic data

The previous study of Dreisigacker et al. (2021) for evaluating pedigree and genomic prediction using 52,242 CIMMYT wheat lines comprised data from the years 2014 through 2019. The dataset in this study incorporates data from two more years of evaluations (2020 and 2021). This dataset includes grain yield records for a total of 68,836 wheat lines that were evaluated at the Norman E. Borlaug Experimental Station in Ciudad Obregon, Mexico, under optimal field management conditions during eight cycles (2014–2021). Original data from each year make up a large number of the trials (200–300) where each trial is comprised of a total of 30 wheat lines established in an alpha‐lattice design of five incomplete blocks of size six lines with three replicates. Phenotypes were corrected by the experimental design by calculating the best linear unbiased estimates of the lines within year. The basic model for each year included an intercept, the random effects of trials, the random effects of the replicates within trials, the random effects of the incomplete blocks within trials and replicates, and the fixed effects of the breeding lines within trials. The corrected grain yield was obtained as the intercept plus the effect of the line.

### 2.2 Genotype data

The genotypic information consisted of 11,293 GBS markers for all lines. Genotyping was performed using the GBS method (see Poland et al. [2012] and Glaubitz et al. [2014] for more details) with lines sequenced using an Illumina HiSeq2500 sequencer at Kansas State University. Marker polymorphisms were called with TASSEL (https://tassel.bitbucket.io) v5.0 and the GBS pipeline (Glaubitz et al., 2014) v2. We filtered markers by keeping only those with <30% of missing values. The markers passing this filter were imputed using the observed allelic frequencies. After imputing, we removed markers with a minor allelic frequency <0.05. After quality control and imputation, a total of 6,978 markers were available for predictions.

### 2.3 Genomic prediction models and methods

We used the GBLUP as a baseline model for genomic prediction of grain yield. Next, we used the SSI as an extension of the GBLUP to obtain a sparse ‘Hat’ matrix from which predictions were derived. Finally, we trimmed the TRN by discarding training individuals with the lowest proportion of nonzero values in the sparse Hat matrix.

### 2.4 GBLUP model

In the GBLUP, the phenotype of the response (grain yield) for the *i*th individual *y_i_* (*i* = 1, 2, …, *n*) is modeled as the sum of its genetic value *u_i_* plus a residual term ε*_i_* as follows:

(1) $$y_{i}=u_{i}+\varepsilon_{i}$$

where the genetic and residual are considered random variables. Vectors **u** = {*u_i_*} and **ε** = {ε*_i_*} are assumed to be normally distributed as $u∼MVN(0,\sigma_{u}^{2}G)$ and $\varepsilon ∼MVN(0,\sigma_{\varepsilon }^{2}I)$, respectively, where $\sigma_{u}^{2}$ is the genetic variance, **G** is an additive genetic relationship matrix, $\sigma_{\varepsilon }^{2}$is the residual variance, and **I** is an identity matrix. The response was previously centered and scaled within year (by subtracting the year sample mean to each observation and then dividing the resulting quantity by the year standard deviation), therefore, no fixed effects for year nor intercept were considered.

The predicted genetic values for all subjects in a prediction (or testing, TST) set are ${\hat{u}}_{\mathrm{TST}}=\left\{{\hat{u}}_{i}\right\}\left(i=1,2,\text{…},n_{\mathrm{TST}}\right)$. These predictions are simply regressions on the observations from the training (TRN) data as ${\hat{u}}_{\mathrm{TST}}=\hat{B}y_{\mathrm{TRN}}$. The matrix $\hat{B}=\left\{{\hat{b}}_{ij}\right\}\left(i=1,2,\text{…},n_{\mathrm{TST}},j=1,2,\text{…},n_{\mathrm{TRN}}\right)$ is the so‐called Hat projection matrix with dimensions *n*_TST_ × *n*_TRN_, containing, at the *i*th row, estimates of the regression coefficients on all *n*_TRN_ training subjects, ${\hat{b}}_{i}=\left[{\hat{b}}_{i1},{\hat{b}}_{i2},\text{…},{\hat{b}}_{in_{\mathrm{TRN}}}\right]$, when predicting the genetic value of the *i*th testing individual. This matrix is given by the following:

(2) $$\hat{B}=G_{\mathrm{TST},\mathrm{TRN}}{\left(G_{\mathrm{TRN}}+\theta I\right)}^{-1}$$

where **G**_TST,TRN_ is the *n*_TST_ × *n*_TRN_ matrix containing the genetic relationships between predicted subjects and those in the TRN, **G**_TRN_ is the genetic relationship matrix of the training data, and $\theta =\sigma_{\varepsilon }^{2}/\sigma_{u}^{2}$ is the ratio between residual and genetic variances.

### 2.5 Sparse selection index

This approach combines a sparsity‐inducing technique with the selection index theory. In the SSI, the regression coefficients for the *i*th predicted individual (**b** *_i_* in Equation 2) are subjected to sparsity by considering a penalized version of the selection index optimization problem as follows:

(3) b∼i=argminbi12b′iGTRN+θIbi−GTST(i),TRNbi+12λbi1

where **G**_TST(_ *_i_*_),TRN_ is the *i*th row vector of the matrix **G**_TST,TRN_; λ is a sparsity‐controlling parameter; and ${∥b_{i}∥}_{1}=\sum_{j=1}^{n_{\mathrm{TRN}}}|b_{ij}|$ is an L1‐penalty on the coefficients **b** *_i_*. The sparse Hat matrix is then $\tilde{B}=\left\{{\tilde{b}}_{ij}\right\}$ and contains, at the *i*th row, the vector ${\tilde{b}}_{i}$ with the solutions to the above‐mentioned problem. When λ = 0, the resulting matrix is equal to that of the GBLUP model, that is, $\tilde{B}=\hat{B}$. A value of λ > 0 produces a sparse Hat matrix with which training individuals contribute to the prediction of some but not all individuals in the TST.

### 2.6 Trimmed sparse selection index

An extra trimming process was added to the original SSI method to completely remove less important training individuals for prediction. We will refer as the ‘trimmed SSI’ (TSSI) to the SSI with the trimmed TRN to distinguish it from the original SSI application.

We trimmed the TRN by zeroing out complete columns from the sparse Hat matrix (with dimensions *n*_TST_ × *n*_TRN_) that have a certain frequency of nonzero values, $F_{\mathrm{TRN}(j)}=\sum_{i=1}^{n_{\mathrm{TST}}}1(b_{ij}\neq 0)$, where 1( · ) is the indicator function that returns the value 1 if $b_{ij}\neq 0$ and 0 otherwise. We discarded the *j*th training element whose value *F*_TRN(_ *_j_*_)_ was smaller than the quantile *Q_p_* of the distribution of *F*_TRN(_ *_j_*_)_ and performing this task for quantiles between *p* = .05 and *p* = .8 with steps of .05. The resulting SSI (TSSI) obtained with the trimmed sparse Hat matrix was denoted as TSSI_1–_ *_p_*. The original SSI using the untrimmed sparse Hat matrix is equivalent to the TSSI_1.0_ where no training individuals are dropped. The importance of the TSSI is that the discarded training individuals do not contribute to the prediction of any predicted individuals. Note that despite the fact that the TSSI method can optimize the TRN by removing the distantly related individuals, the genotypes are still required for these distantly related individuals (trimmed individuals) when computing the sparse Hat matrix before applying the trimming process.

### 2.7 Training and TSTs

The TSTs were composed of cycles TST = 2019, 2020, and 2021 separately. Five different TRNs were considered for each TST. First, TRN_1_ was composed of individuals from 1 yr before the TST, and then the TRNs were formed by accumulating 2, 3, 4, or 5 previous years (denoted as TRN_1–_ *_k_*, k = 2, 3, 4, 5). For example, to predict TST = 2021 genotypes, the following sets were used: TRN_1_ = 2020, TRN_1–2_ = 2020 + 2019, …, TRN_1–5_ = 2020 + … +2016 (Table 1).

**TABLE 1:** Size of the training set (*n*_TRN_) used for each prediction set (TST)

TRN	TST (*n*_TST_)		
	2021 (7,893)	2020 (8,701)	2019 (8,928)
TRN_1_	8,701	8,928	8,310
TRN_1–2_	17,629	17,238	17,702
TRN_1–3_	25,939	26,630	26,974
TRN_1–4_	35,331	35,902	35,908
TRN_1–5_	44,603	44,836	43,314

*Note*. TRN, training sets composed of one cycle previous to the TST (TRN_1_), and cumulative sets (TRN_1–_ *_k_*) formed with the closest *k* (*k* = 2, 3, 4, or 5) cycles before the TST.

A genomic relationship matrix was calculated as $G={\mathrm{XX}}^{\prime }/p$ (Lopez‐Cruz et al., 2015), where **X** is the matrix containing the *p* = 6,978 biallelic centered and standardized markers. Genetic models (as in Equation 1) were fitted to grain yield within each TST‐TRN combination to estimate variance components ($\sigma_{\varepsilon }^{2}$ and $\sigma_{u}^{2}$) from which the variances ratio and genomic heritability were estimated as $\hat{\theta }={\hat{\sigma }}_{\varepsilon }^{2}/{\hat{\sigma }}_{u}^{2}$ and ${\hat{h}}_{g}^{2}={\hat{\sigma }}_{u}^{2}/\left({\hat{\sigma }}_{\varepsilon }^{2}+{\hat{\sigma }}_{u}^{2}\right)$ , respectively. These estimates were used to derive non‐sparse and sparse Hat matrices (Equation 2 and 3). For the sparse Hat matrix, an extra step was required to calculate an optimal value of the penalization λ (Equation 3). A trimmed sparse Hat matrix was then obtained by zeroing out complete columns from the sparse Hat matrix (see previous section). The genetic value predictions (${\hat{u}}_{\mathrm{TST}}$) for the prediction cycle with the GBLUP, SSI, and TSSI were computed using the nonsparse, sparse, and trimmed sparse Hat matrices, respectively.

Prediction accuracy was evaluated within each prediction cycle as the Pearson correlation between observed and predicted values, $\mathrm{cor}(y_{\mathrm{TST}},{\hat{u}}_{\mathrm{TST}})$. The prediction performance was also assessed using the mean squared error as $\mathrm{MSE}=\frac{1}{n_{\mathrm{TST}}}{\sum_{i=1}^{n_{\mathrm{TST}}}\left[y_{\mathrm{TST}(i)}-{\hat{u}}_{\mathrm{TST}(i)}\right]}^{2}$.

### 2.8 Optimizing the penalization parameter

An optimal value of the penalization parameter for the SSI (in Equation 3) was found by internal cross‐validation as described in Lopez‐Cruz et al. (2021). The procedure consists of using each TRN for optimizing the penalized parameter under different prediction cases using years 2019, 2020, and 2021 separately as TSTs and the previous years used as TRN. Therefore, within each TRN, we performed 10‐fold cross‐validation as follows. First, training data was divided into 10 subsets (folds), then, a value of MSE was computed for each value of λ in a grid of 100 decreasing λ values (evenly spaced in the logarithm scale and ranging from the maximum possible to near zero, λ_max_ = λ_1_ > λ_2_ > … > λ_100_ = 1 × 10^−7^, where $\lambda_{\max}=\max_{j}\{\frac{|G_{\mathrm{TST}(i),\mathrm{TRN}(j)}|}{\sqrt{G_{\mathrm{TRN}(j),\mathrm{TRN}(j)}+\hat{\theta }}}\}$) for each fold using the remaining nine folds for model training. The procedure was repeated for five different 10‐fold partitions of the training data. Finally, the optimal value was chosen as the one that minimized the average MSE curve across the 5 × 10 = 50 folds.

### 2.9 Software

All analyses were performed in R v4.0.3 (R Core Team, 2020). The genomic matrix was obtained using the ‘getG’ function from the BGData package (Grueneberg & de los Campos, 2019), whereas variance components were estimated using the function ‘fitBLUP’ from the SFSI package (Lopez‐Cruz et al., 2020). The regression coefficients for the SSI models were computed with the function ‘SSI’ from the SFSI package. All analyses were implemented using high‐performance computing resources from Michigan State University (https://icer.msu.edu/hpcc/hardware).

## 3 RESULTS

### 3.1 Genomic relationships and heritabilities within TRNs

Out‐diagonal genomic values (*g_ij_*) in the **G** matrix corresponding to the individuals in the prediction (*i* = 1, 2, …, *n*_TST_) and training (*j* = 1, 2, …, *n*_TRN_) sets TRN_1–5_ are provided in Supplemental Figures S1–S3 for the prediction years TST = 2019, 2020, and 2021, respectively. These values show similar distributions across all the training years forming the TRN_1–5_ set with a no significant decay in the quantiles but in the rank of the distribution with more years apart. In general, all years in the data set (2014–2021) are interconnected with each other. The degree of connectivity varies from one extreme case where genotypes that are distant in time are closely related (according to the coordinates on the top two principal components, Supplemental Figure S4) to the other extreme case where genotypes from two consecutive years are distantly related (based on top principal components). The proportion of variance explained by markers (${\hat{h}}_{g}^{2}$) was between .20 and .38 and decreased as more previous cycles were added to the TRN. For instance, when predicting the TST = 2021 cycle, the genomic heritability obtained with the 2020 cycle (TRN_1_) was ${\hat{h}}_{g}^{2}$ = .34 and was reduced up to .21 when cycles 2016–2020 were combined (TRN_1–5_) in the TRN (Table 2). Similar results were obtained for the prediction of TST = 2020 (${\hat{h}}_{g}^{2}$ = .32 with TRN_1_ and .21 with TRN_1–5_) and TST = 2019 (${\hat{h}}_{g}^{2}$ = .39 with TRN_1_ and .21 with TRN_1–5_).

**TABLE 2:** Prediction accuracy and mean squared error for each training set (TRN) composition for grain yield prediction at each cycle in 2019, 2020, and 2021

					Accuracy			MSE		
TST	TRN	${\hat{\sigma }}_{u}^{2}$	${\hat{\sigma }}_{\varepsilon }^{2}$	${\hat{h}}_{g}^{2}$	GBLUP	SSI	Pr^a^	GBLUP	SSI	Pr^b^
2021	1	0.33	0.64	0.34	0.259	0.266	0.9336	0.349	0.339	1.0000
	1–2	0.26	0.71	0.27	0.268	0.285	1.0000	0.336	0.328	1.0000
	1–3	0.25	0.72	0.25	0.271	0.291	1.0000	0.343	0.332	1.0000
	1–4	0.21	0.73	0.22	0.295	0.305	0.9906	0.335	0.328	1.0000
	1–5	0.19	0.74	0.21	0.307	0.315	0.9770	0.329	0.326	0.9958
2020	1	0.32	0.67	0.32	0.318	0.324	0.9680	0.308	0.300	1.0000
	1–2	0.27	0.70	0.28	0.363	0.356	0.0228	0.295	0.293	0.9260
	1–3	0.22	0.72	0.23	0.366	0.358	0.0236	0.292	0.291	0.7918
	1–4	0.20	0.73	0.22	0.356	0.351	0.1150	0.295	0.293	0.9474
	1–5	0.19	0.74	0.21	0.365	0.371	0.9304	0.292	0.288	1.0000
2019	1	0.38	0.61	0.39	0.296	0.305	0.9976	0.418	0.403	1.0000
	1–2	0.25	0.67	0.27	0.299	0.305	0.9720	0.411	0.400	1.0000
	1–3	0.22	0.69	0.24	0.332	0.337	0.9538	0.397	0.388	1.0000
	1–4	0.21	0.72	0.22	0.346	0.346	0.5484	0.391	0.384	1.0000
	1–5	0.19	0.72	0.21	0.347	0.345	0.2970	0.392	0.385	1.0000

*Note*. TST, prediction set; TRN, training sets composed of one previous cycle to the TST (TRN_1_), and cumulative sets (TRN_1–k_) formed with the closest k (k = 2, 3, 4, or 5) cycles before the TST; MSE, mean squared error; GBLUP, genomic best linear unbiased prediction; SSI, sparse selection index; ${\hat{\sigma }}_{u}^{2}$, estimated genetic variance; ${\hat{\sigma }}_{\varepsilon }^{2}$, estimated residual variance; ${\hat{h}}_{g}^{2}$, estimated genomic heritability. Pr^a^, proportion of times that the bootstrapped accuracy of the SSI was greater than that of the GBLUP; Pr^b^, proportion of times that the bootstrapped MSE of the SSI was smaller than that of the GBLUP. Bootstrap was carried out by subsampling with replacement 5,000 times predicted and observed grain yield values.

### 3.2 Historical data adds accuracy for prediction depending on TRN size

Adding *k* > 1 yr of previous cycles in the TRN (TRN_1‐_ *_k_*) represented a growth of the training size of about *k*‐folds as opposed to using only one previous year as training (TRN_1_). These increases in the training size were reflected in an improved accuracy of the GBLUP models relative to the model trained with TRN_1_ (Table 2). However, when the TRN size was already large, sometimes the prediction accuracy remained unchanged with increasing the number of cycles in the TRN. For the prediction of TST = 2021 genotypes, the accuracy was improved by 3 (with TRN_1–2_), 5 (with TRN_1–3_), 14 (with TRN_1–4_), and up to 19% (i.e., ∼0.05 points in the correlation scale) using the TRN_1–5_ (*n*_TRN_ = 44,603) when compared with the TRN_1_ (*n*_TRN_ = 8,701) case. The improvement in accuracy of TRN_1–5_ to TRN_1_ was of the same magnitude for the TST = 2020 (15%) and TST = 2019 (17%) predictions.

**FIGURE 1:**
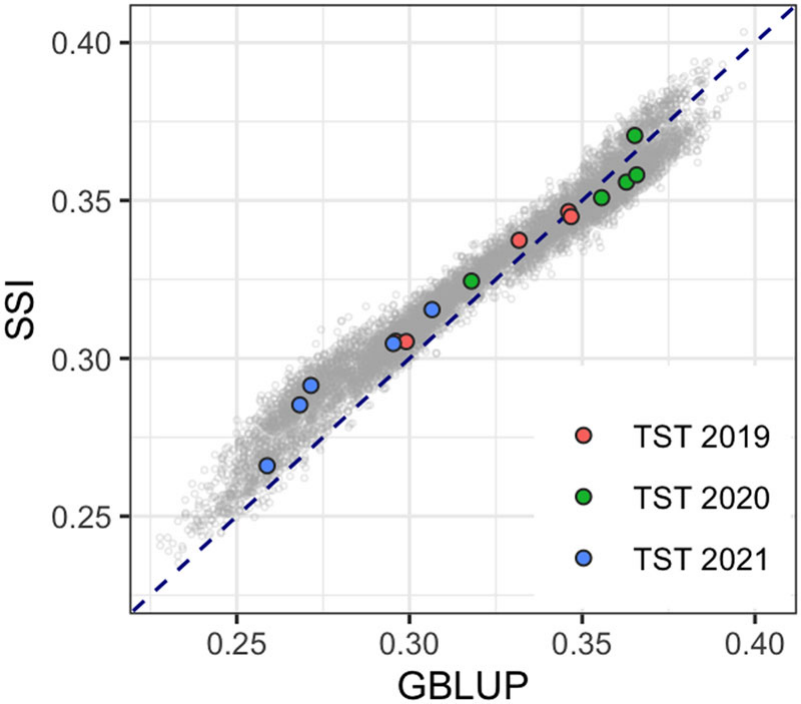
Prediction accuracy of the genomic best linear unbiased prediction (GBLUP) and of the sparse selection index (SSI). Each point represents a prediction case: a prediction (TST) cycle (TST = 2019, 2020, or 2021, separated by color) using the five training (TRN) sets (TRN_1_, TRN_1–2_, …, and TRN_1–5_). Points in gray represent 500 bootstrapped instances for each prediction case. The 45° line is shown for comparison, where points above and below the line has a larger and smaller, respectively, accuracy than the other model

In all TST–TRN cases, the SSI showed a lower MSE than the GBLUP (Supplemental Figure S5). However, in almost all cases, the accuracy of the SSI was larger than that of the GBLUP model (Table 2, Figure 1). The largest improvement in accuracy was ∼0.02 (when predicting the TST = 2021 cycle with TRN_1–2_ and TRN_1–3_) with a reduction in MSE of ∼0.01 (see Table 2). Similar to GBLUP, the accuracy of the SSI models improved from increases in the TRN size.

**FIGURE 2:**
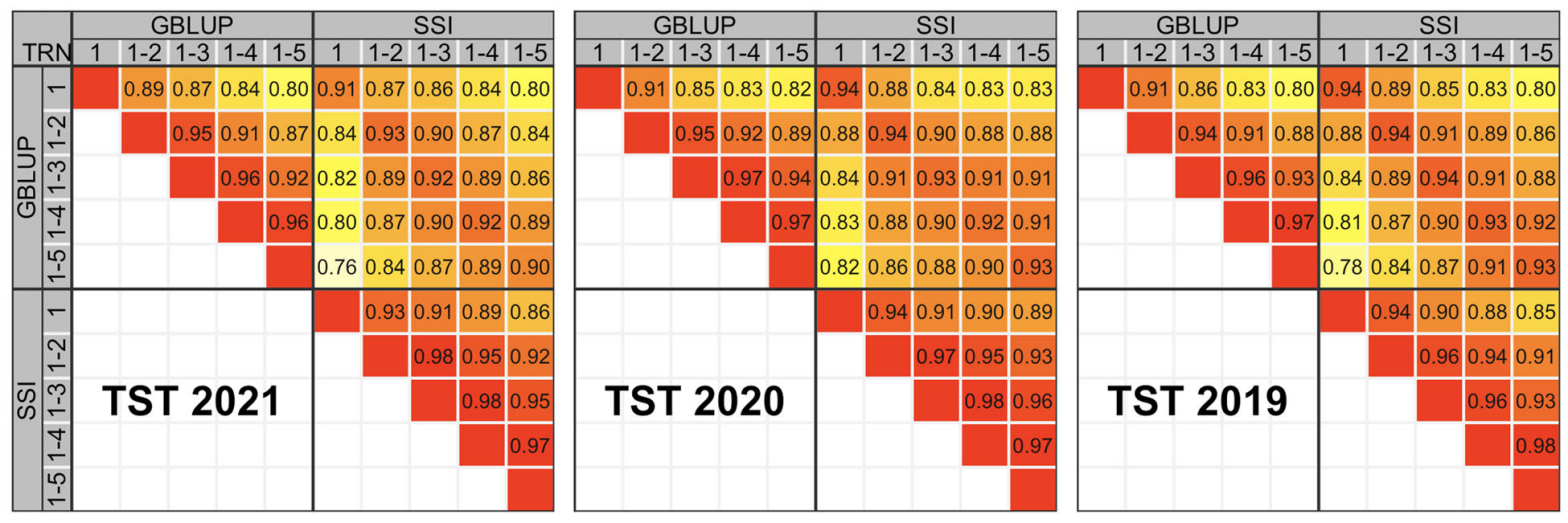
Correlation between predicted values obtained by each model (genomic best linear unbiased prediction [GBLUP] and of the sparse selection index [SSI]) and training (TRN) set (TRN_1_, TRN_1–2_, …, and TRN_1–5_). Each panel represents a prediction (TST) cycle (TST = 2019, 2020, or 2021)

Although yielding slightly more accurate predictions, in general, the predicted values obtained with the SSI are very similar to those of the GBLUP models (correlation between 0.90 and 0.94, see Figure 2). The correlation between predicted values obtained with the GBLUP model when using one and two previous cycles (TRN_1_ and TRN_1–2_) is between 0.89 and 0.91 (Figure 2), which decayed when compared with TRN_1_, as more previous cycles were included in the TRN (between 0.85 and 0.87 with TRN_1–3_ and up to 0.80–0.82 with TRN_1–5_). However, with the SSI model, the predictions with TRN_1_ were very similar to those obtained with TRN_1–2_ (correlation of 0.93–0.94) and are still in substantial agreement as more previous cycles were added to the TRN relative to when using TRN_1_ (correlation of 0.90–0.91 with TRN_1–3_ and 0.85–0.89 with TRN_1–5_). These results (Figure 2) suggest that the SSI provides a more stable ranking of genotypes than the GBLUP as the TRN becomes larger.

### 3.3 Inspection of the Hat matrix to assess predictive contribution

**FIGURE 3:**
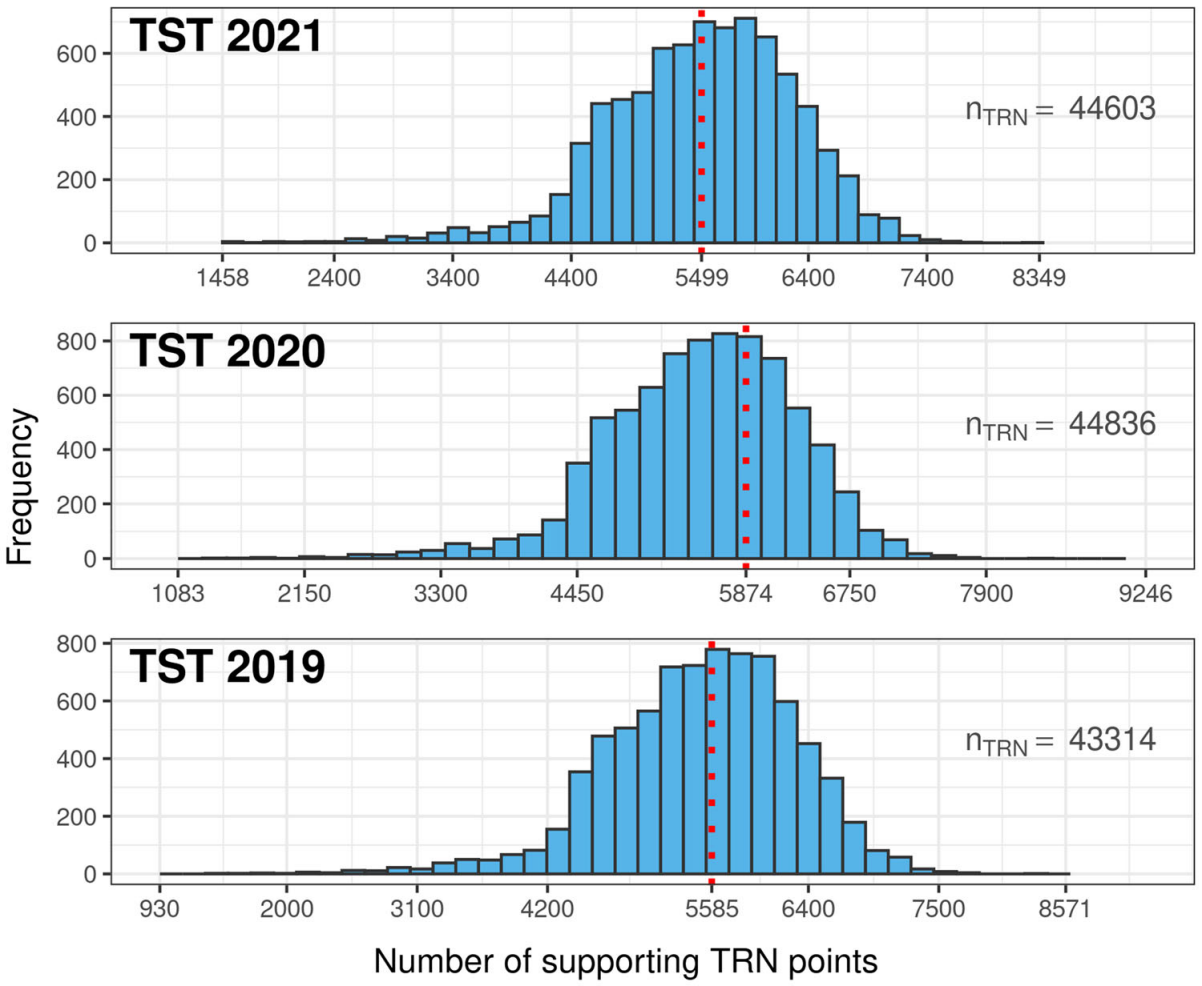
Frequency of the number of supporting training (TRN) points for each individual in the prediction (TST) cycle (TST = 2019, 2020, or 2021), $F_{TST(i)}=\sum_{j=1}^{n_{TRN}}1\left(b_{ij}\neq 0\right)$, where 1( · ) is the indicator function that returns the value 1 if $b_{ij}\neq 0$ and 0 otherwise (i.e., number of nonzero values at each row of the sparse Hat matrix). The TRN set was composed of the last five cycles before the TST set (TRN_1‐5_). Vertical dotted lines represent the mean value

In the SSI, the *i*th predicted subject (*i* = 1, 2, …, *n*_TST_) receives prediction support from some but not all the training individuals. This number, $F_{TST(i)}=\sum_{j=1}^{n_{TRN}}1\left(b_{ij}\neq 0\right)$, is given by the frequency of nonzero *b_ij_* coefficients at each row of the sparse Hat matrix. Figure 3 shows the distribution of the *F*_TST(_ *_i_*_)_ values for each TST (TST = 2019, 2020, and 2021) using the last 5 yr (TRN_1–5_) to train the model. The TRN_1–5_ is composed of >40,000 individuals that within the GBLUP model provide prediction to all predicted genotypes (i.e., *F*_TST(_ *_i_*_)_ = *n*_TRN_ for all *i*). The SSI formed the prediction of TST = 2021 genotypes using between 1,458 < *F*_TST(_ *_i_*_)_ < 8,349 training genotypes, with an average of ${\bar{F}}_{\mathrm{TST}}$ = 5,499 training elements. That is, the prediction of the individuals in TST = 2021 is provided by, on average, only 12% of the total *n*_TRN_ = 44,603 elements available in TRN_1–5_ (see the top panel in Figure 3). Similarly, the TST = 2020 and TST = 2019 predictions of genotypes were performed with ${\bar{F}}_{TST}=$5,874 (out of *n*_TRN_ = 44,836) and ${\bar{F}}_{\mathrm{TST}}$ = 5,585 (out of *n*_TRN_ = 43,314) training individuals, respectively.

**FIGURE 4:**
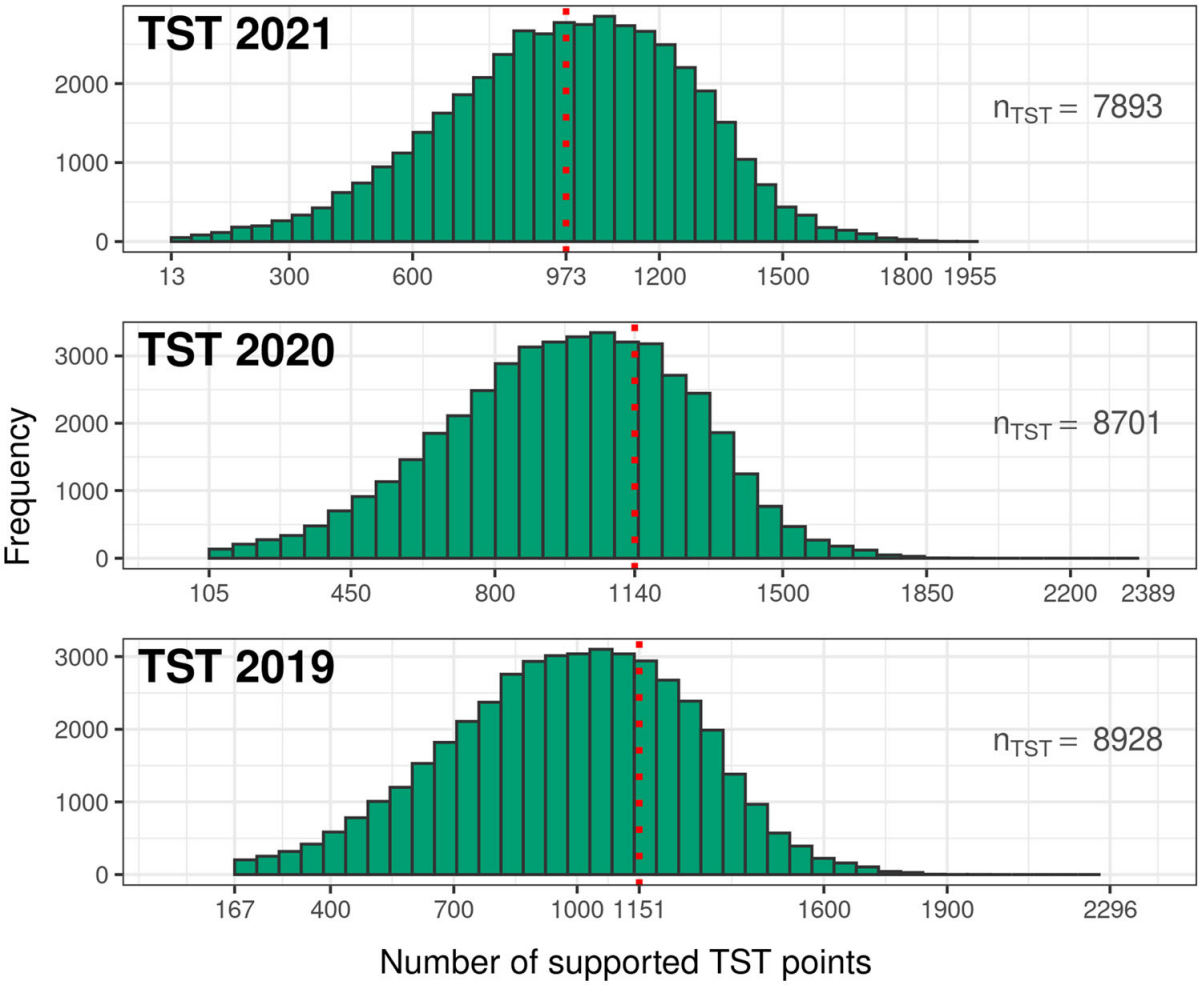
Frequency of the number of supported points in the prediction (TST) cycle (TST = 2019, 2020, or 2021) of each training (TRN) individual, $F_{\mathrm{TRN}(j)}=\sum_{i=1}^{n_{\mathrm{TST}}}1(b_{ij}\neq 0)$, where 1( · ) is the indicator function that returns the value 1 if $b_{ij}\neq 0$ and 0 otherwise (i.e., number of nonzero values at each column of the sparse Hat matrix). The TRN set was composed of the last five cycles before the TST set (TRN_1–5_). Vertical dotted lines represent the mean value

Upon inspecting the columns of the sparse Hat matrix, $F_{\mathrm{TRN}(j)}=\sum_{i=1}^{n_{\mathrm{TST}}}1(b_{ij}\neq 0)$ gives the frequency of nonzero *b_ij_* coefficients at each column of the matrix. These *F*_TRN(_ *_j_*_)_ values represent the number of predicted individuals to which a single training subject *j* provides prediction. Figure 4 shows the distribution of the *F*_TRN(_ *_j_*_)_ values for each TST (TST = 2019, 2020, and 2021) using the last 5 yr (TRN_1–5_) as a TRN. Training individuals support the prediction of only some individuals in the TST. For instance, in the prediction of the *n*_TST_ = 7,893 genotypes in TST = 2021, training individuals were supporting the prediction of a reduced number of subjects as low as *F*_TRN(_ *_j_*_)_ = 13 and up to *F*_TRN(_ *_j_*_)_ = 1,955 genotypes. On average, individuals in TRN_1–5_ supported the prediction for only ${\bar{F}}_{\mathrm{TRN}}$ = 973 individuals in TST = 2021 (see the top panel in Figure 4). In the TST = 2020 and TST = 2019 prediction cycles, individuals in TRN_1–5_ provided predictions for only ${\bar{F}}_{\mathrm{TRN}}$ = 1,140 (out of *n*_TST_ = 8,701) and to ${\bar{F}}_{\mathrm{TRN}}$ = 1,151 (out of *n*_TST_ = 8,928) predicted genotypes, respectively.

### 3.4 Trimming the TRN supports optimization of accuracy

**FIGURE 5:**
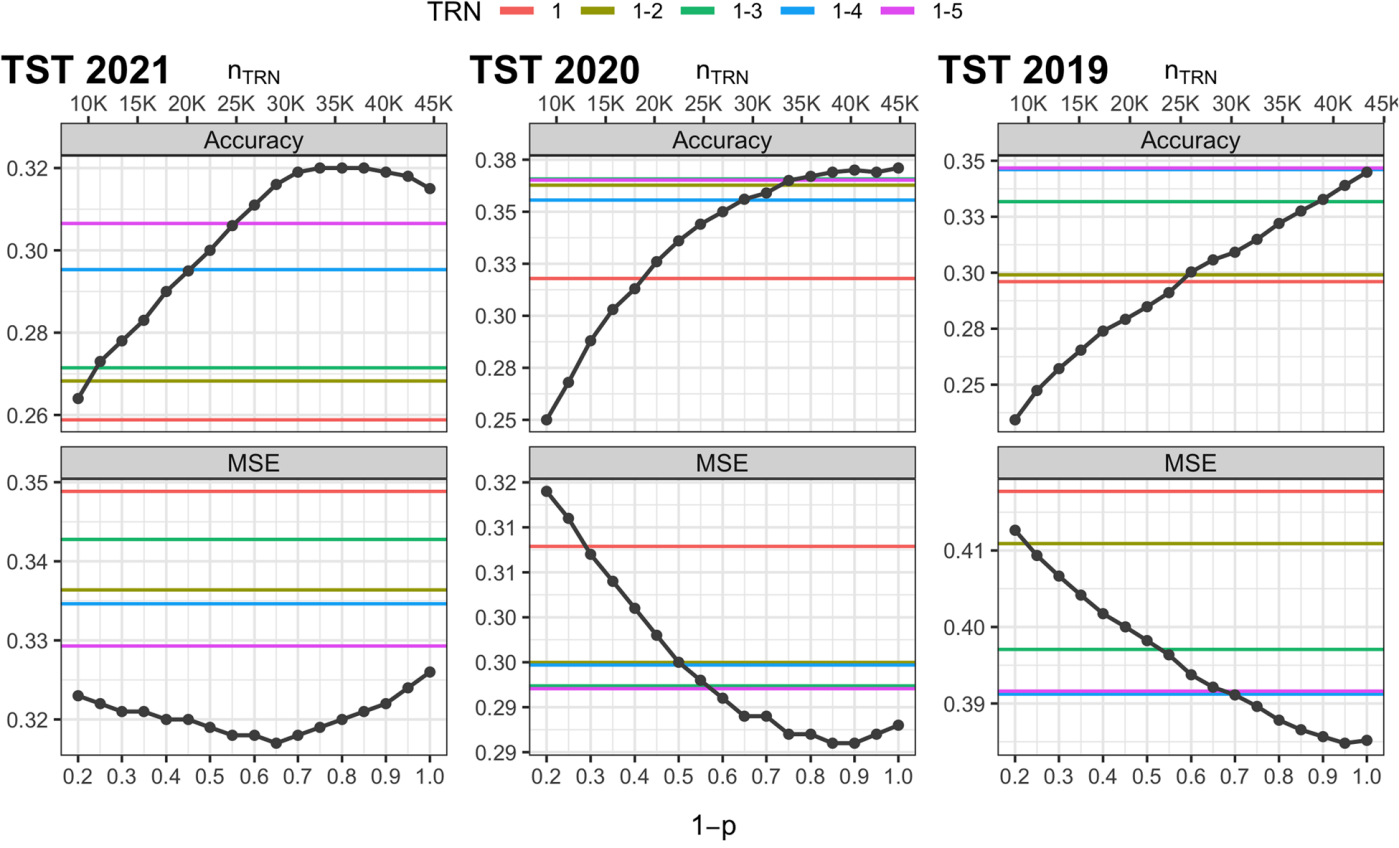
Prediction accuracy and mean square error (MSE; black lines) of the TSSI_1–_ *_p_* model for each prediction (TST) cycle (TST = 2019, 2020, or 2021). The predictions are obtained by using a reduced training (TRN) data TRN_1–5_ (the last five previous cycles to the TST). Individuals in TRN_1–5_ whose frequency *F*_TRN(_ *_j_*_)_ was smaller than the quantile *Q_p_* (*p* = .05, .10, .15, …, .75, and .80) were dropped from the sparse Hat matrix; and an index TSSI_1–_ *_p_* was calculated with the trimmed sparse Hat matrix. The index TSSI_1.0_ at the right‐most side of the plots correspond to the original sparse selection index fitted with all TRN_1–5_ data. The top *x* axis shows the reduced TRN set size associated to each TSSI_1–_ *_p_* model. Horizontal colored lines represent the accuracy and MSE of the GBLUP model fitted with each TRN set (TRN_1_ and cumulated training TRN_1‐_ *_k_*, *k* = 2, 3, 4, or 5)

Zeroing out complete columns of the sparse Hat matrix, whose value *F*_TRN(_ *_j_*_)_ was smaller than a certain threshold, resulted in a reduction of the total TRN. In the prediction of TST = 2021 genotypes, the accuracy of the SSI obtained with the whole TRN_1–5_ (*n*_TRN_ = 44,603) was 0.315 with an MSE of 0.326. Trimming the whole TRN_1–5_ data, by discarding individuals whose value *F*_TRN(_ *_j_*_)_ < *Q*_0.35_, resulted in a TSSI_0.65_ using a smaller TRN (*n*_TRN_ = 29,032) still containing individuals from all training cycles at comparable frequencies (6,487 subjects from cycle TRN = 2020; 5,016 from TRN = 2019; 4,244 from TRN = 2018; 6,774 from TRN = 2017; and 6,511 from TRN = 2016, see Table 3). This reduced TRN, which represented 65% of the total TRN_1–5_, yielded the same prediction accuracy (∼0.316) of the SSI trained with the whole TRN_1–5_ but with a minimized MSE (0.317, see Figure 5). When dropping almost half of the TRN_1–5_ data (*F*_TRN(_ *_j_*_)_ < *Q*_0.45_, *n*_TRN_ = 24,580), the resulting index TSSI_0.55_ yielded the same accuracy (∼0.306) but with a reduced MSE (0.318) relative to the GBLUP (accuracy = 0.307, MSE = 0.329) trained with all data TRN_1–5_.

**TABLE 3:** Number of training (TRN) individuals by cycle used to predict grain yield in 2019, 2020, and 2021 data for each reduced TRN data (the total TRN is composed of the last five cycles before the prediction cycle)

TST	TRN	1‐proportion of the subjects dropped from the TRN										
		1.0	0.95	0.90	0.85	0.80	0.75	0.70	0.65	0.60	0.55	0.50
2021	2020	8,701	8,322	7,975	7,671	7,385	7,064	6,775	6,487	6,172	5,869	5,551
	2019	8,928	8,230	7,620	7,042	6,524	5,998	5,529	5,016	4,571	4,078	3,604
	2018	8,310	7,658	7,026	6,424	5,850	5,285	4,735	4,244	3,794	3,372	2,983
	2017	9,392	9,067	8,718	8,374	7,983	7,590	7,183	6,774	6,307	5,832	5,313
	2016	9,272	9,108	8,803	8,403	7,959	7,518	7,004	6,511	5,982	5,429	4,866
	Total	44,603	42,385	40,142	37,914	35,701	33,455	31,226	29,032	26,826	24,580	22,317
2020	2019	8,928	8,388	7,927	7,534	7,215	6,902	6,569	6,286	5,972	5,635	5,308
	2018	8,310	7,679	7,155	6,676	6,248	5,813	5,417	5,032	4,645	4,267	3,873
	2017	9,392	8,905	8,445	7,956	7,409	6,883	6,321	5,751	5,227	4,695	4,140
	2016	9,272	8,875	8,340	7,770	7,223	6,643	6,073	5,502	4,970	4,425	3,897
	2015	8,934	8,760	8,503	8,189	7,811	7,424	7,005	6,572	6,124	5,651	5,232
	Total	44,836	42,607	40,370	38,125	35,906	33665	31,385	29,143	26,938	24,673	22,450
2019	2018	8,310	7,813	7,481	7,186	6,886	6610	6,364	6,047	5,756	5,445	5,118
	2017	9,392	8,780	8,231	7,699	7,193	6667	6,157	5,702	5,253	4,776	4,300
	2016	9,272	8,741	8,187	7,629	7,116	6608	6,120	5,577	5,074	4,541	4,067
	2015	8,934	8,592	8,240	7,875	7,433	7006	6,553	6,139	5,662	5,231	4,764
	2014	7,406	7,222	6,860	6,440	6,039	5613	5,134	4,708	4,281	3,837	3,431
	Total	43,314	41,148	38,999	36,829	34,667	32,504	30,328	28,173	26,026	23,830	21,680

*Note*. TST, prediction set; TRN, training set cycle that formed the total TRN_1–5_ (the last five cycles before the TST). Reduced TRN used in the obtention of TSSI_1–_ *_p_* derived by dropping the *p* × 100% of the TRN_1–5_; that is, individuals in TRN_1‐5_ whose frequency *F*_TRN(_ *_j_*_)_ < *Q_p_*, for *p* = .05, .10, .15, …, .45, and .50). The index TSSI_1.0_ corresponds to the original sparse selection index (SSI) fitted with all TRN_1–5_ data.

In the case of the TST = 2020 prediction, a TSSI_0.75_ (i.e., dropping the quantile 25% from TRN_1–5_) yielded the same accuracy (0.365) and a slightly smaller MSE (0.287) than the GBLUP (MSE = 0.292) trained with TRN_1–5_ (Table 2, Figure 5). Although no benefit was observed in terms of accuracy in the prediction of TST = 2019 for the proposed trimmed SSI approach, a lower MSE (<0.391) was observed when we drop up to 30% of the training data compared with the GBLUP trained with TRN_1–5_ (MSE = 0.392) or TRN_1–4_ (MSE = 0.391; Table 2, Figure 5). In general, the observed trends were not completely similar for the three prediction cycles.

## 4 DISCUSSION

### 4.1 The complexity of factors involved in genomic‐based predictions

The complexity of the genomic‐enabled prediction is evident and shown in terms of several factors including sample size, genotype × environment interaction, trait heritability, LD between markers and quantitative trait loci, family relationships between individuals in training and prediction groups, and diversity within the TRN (Daetwyler et al., 2008; Heffner et al., 2009; Lorenzana & Bernardo, 2009; Combs & Bernardo, 2013; Crossa et al., 2017).

As pointed out by Habier et al. (2007), the relationship between the several sources of accuracy produced by the above‐mentioned factors is not easy to untangle and difficult to study in isolation. For example, increasing marker density can increase the proportion of the trait variance explained by GBLUP models but requires markers in LD with quantitative trait loci in both TRNs and TSTs. Lopez‐Cruz et al. (2021) showed that accuracy increased when increasing the training size by adding only a few individuals in the same cycle; however, the genomic heritability remained unchanged. This suggests a sizable contribution of closely related individuals (i.e., individuals in the same cycle) to increase prediction accuracy. Lopez‐Cruz et al. (2021) pointed out that these results agree with Habier et al. (2007) in the sense that the accuracy of GBLUP models mostly results from the genetic relationships between individuals in training and prediction groups rather than the existing LD.

Increasingly heterogeneous populations with a high degree of family structure can make genetic effects vary across subgroups (de los Campos et al., 2015b). Therefore, the classical GBLUP model that assumes homogeneous genetic effects may be less able to capture overall genetic patterns, thus reducing the proportion of the additive variance explained by markers. In our study, increasing the training size (and therefore, increasing genetic diversity and heterogeneity) by including more years in the TRN was reflected in an improvement in prediction accuracy; however, this also implied a reduction of the genomic heritability (Table 2). The prediction accuracy depends on the interplaying of the narrow‐sense heritability (proportion of the trait variance explained by additive genetic effects) with the accuracy of the estimated genetic effects (proportion of additive variance explained by markers) (e.g., Goddard, 2009; de los Campos et al., 2015a). Therefore, our results suggest that the gains in the GBLUP accuracy resulted predominately from using a large TRN (potentially including closely related individuals to the ones to be predicted) with a broad genetic basis rather than in increasing additive variance captured by markers (i.e., LD). These observations are also in agreement with findings in Makowsky et al. (2011) and Habier et al. (2007).

### 4.2 Influence of assessing year × genotype interaction

Because of the large number of lines evaluated each year in this study, GBLUP models including genotype × environment interaction were not deployed. When using actual data from a breeding program, most of the breeding lines evaluated in one year are not repeated the following year and thus interaction terms between genetic (marker) information and environments can only be assessed by the link between individuals established by the markers. Thus, it is possible to borrow information for predicting unobserved lines in new environments by using markers and correlated environmental information. However, it must be accepted that some degree of confounding information between genomic and environments exist in large plant breeding trials when not all lines are tested in all environments and the degree of overlapping of lines across years (or environments) is low. In the Pérez‐Rodríguez et al. (2017) study, when using a large number of CIMMYT lines tested in multienvironment trials (*n* = 58,798), the single‐step genomic and pedigree genotype × environment models yielded 24–66% accuracy gains over models that did not account for genotype × environment interactions. Pérez‐Rodríguez et al. (2020) found that under the statistical reaction norm model including year × genotype interactions (Jarquín et al., 2014), the prediction accuracy based on markers and pedigree was 0.437 for TST = 2018 when training the models using lines from 2014–2017 and was higher that when using either markers or pedigree alone. Using the same models, Dreisigacker et al. (2021) obtained the same prediction accuracy (0.340) as the one obtained in this study for TST = 2019 using 2014–2018 for model training. In general, the authors found slightly higher or similar prediction accuracies than those obtained in previous cycles (years) and those obtained in this study. However, in our study, we did not assess year × genotype interaction and did not include the pedigree information; therefore, the prediction of genotypes relied only on their marker‐derived genetic relatedness with a large number of other genotypes evaluated in a series of one or more previous years.

### 4.3 The impact of individual's distance in TRN and TST on prediction accuracy

Rincent et al. (2012) highlighted that prediction accuracy is increased when the TRN includes individuals distantly related to each other and closely related to those in the TST. Results from this study clearly show that adding more lines from previous years increases the prediction accuracies (Table 2) using the GBLUP and SSI methods. The SSI model demonstrated that a substantial number of historical lines (representing 5 yr in this study) contributed to the prediction of individuals in the prediction year. These results indicate that not all historical breeding lines are increasingly distantly related lines to the current prediction year (Supplemental Figures S1–S4). This can be due to identity by state or the fact that key parents are used in breeding for several years. Because of constant artificial selection, favorable linkage blocks in the genome can be preserved and inherited for several cycles. The SSI showed some superiority over the GBLUP in terms of prediction accuracy by finding the most predictive individuals in the TRN. In general, the contribution to the prediction was not greatly provided by the most recent years, instead the SSI derived the predictions from comparable contributions from all years in the TRN (see Supplemental Figures S6–S8 for plots of genomic relationships vs. regression coefficients).

The CIMMYT wheat data used in this study, as well as those used by Pérez‐Rodríguez et al. (2020, 2017), Howard et al. (2019), and Dreisigacker et al. (2021), comprised a large number of small‐sized families at F_7_ and F_8_ breeding generations and are part of the routine product development pipeline of the CIMMYT spring wheat bread breeding program. The size and the degree of connectivity of the families connecting training and prediction populations vary across years leading to a very complex admixture between families and years (Supplemental Figure S4). Therefore, lines from these small families bred previously can be connected through pedigree with some but not all lines in the more recent cycles, thus increasing the prediction accuracy in the prediction cycle. However, families that are not related to others represent superfluous genetic material for prediction. In this study, the TSSI with a reduced TRN representing 65% of the total TRN_1–5_ data yielded the same prediction accuracy (∼0.316) as the SSI using the total TRN_1–5_ (with a minimized MSE). The TSSI_0.55_ (formed by dropping almost half of the TRN_1–5_ data) also yielded the same accuracy (∼0.306) as that of the GBLUP trained with all data TRN_1–5_ (∼0.307). This reduced TRN was still formed with individuals from all generations equally represented, demonstrating that in the total TRN_1–5_ data, there are individuals (and families) developed 5 yr ago that are still genetically related to those in the TST. It also demonstrated that a great deal of individuals in the TRN do not substantially contribute to the prediction of those in the TST, thus representing redundant information for training. Choosing the most informative lines prescinding of superfluous information in the TRN with the TSSI model can be enough to reach the same prediction accuracy that is obtained using all training data.

Interestingly, the results of this study did not agree with those from Dawson et al. (2013), where, using CIMMYT historical wheat data over 17 yr, the accuracy of year‐to‐year grain yield predictions using TRNs comprising all previous years was approximately the same as when considering only the previous 3 yr. However, the authors obtained these results by sequentially training the model using all data from one or more previous years; with the SSI, all available historical data can be used for model training and let it to automatically perform selection of some but not all training individuals within each year.

In summary, more genetically related individuals in the training and TSTs are detected by SSI methods even when they are not closely related in time. The SSI methodology considers both complexities: (a) the relationships between the genotype in the TST and each genotype in the TRN as well as (b) the relationships between the training genotypes. Therefore, even with a very large TRN, the SSI method should be still able to extract information from those nonredundant individuals more genetically related to those on the TST. The results of the SSI will be different from what can be obtained by selecting training individuals based on thresholding genomic relationships (Lopez‐Cruz & de los Campos, 2021). In our results, training individuals with a sizable genomic relationship with testing individuals are less likely to be included in the optimal TRN (i.e., *b_ij_* ≠ 0) if they are related (i.e., have a relatively high genomic *R* ^2^ coefficient) with other individuals in the TRN (Supplemental Figures S6–S8). Results from this study show that the use of all available multigeneration information together does not decrease the prediction accuracy of the standard GBLUP. However, as demonstrated by Lopez‐Cruz and de los Campos (2021), the SSI provided higher gains in accuracy when compared with the GBLUP, as more years were included in the TRN. Although the amount of additive variance captured by markers decreased, and thus, expecting a reduction of the genomic prediction accuracy, using a very diverse and large enough TRN appeared to be a more influential factor that caused the accuracy to increase. The SSI is useful for TRN optimization to extract core information for prediction with large multigeneration data. Genomic relationships may be complemented with pedigree information to account for polygenic variance not captured by markers, and therefore, potentially benefit the prediction accuracy (Velazco et al., 2019).

## 5 CONCLUSIONS

This study demonstrates that the SSI optimizes prediction accuracy when the training data has complex relationship patterns arising from multiple‐year breeding data. In this context, differences in allele frequencies and in LD patterns may make genetic effects heterogenous across families and subfamilies, thus making the standard GBLUP suboptimal. With the large multiple‐year data used in this study, comprised of wheat grain yield of 68,836 lines generated across 8 yr, small improvements of up to ∼0.05 in the GBLUP accuracy were achieved when using 5 yr of data compared with when using only one previous year. The SSI showed a slight gain over the GBLUP accuracy with the advantage that trimming the SSI allows for these accuracies to be achieved with a minimized MSE using only a portion of the total TRN.

## AUTHORS CONTRIBUTION

Marco Lopez‐Cruz: Formal analysis, Investigation, Methodology, Software, Validation, Writing – original draft, Writing – review & editing. Susanne Dreisigacker: Conceptualization, Writing – original draft. Leonardo Crespo‐Herrera: Resources, Writing – review & editing. Alison R. Bentley: Funding acquisition, Resources, Writing – review & editing. Ravi Singh: Resources, Writing – review & editing. Jesse Poland: Data curation, Resources, Writing – review & editing. Sandesh Shrestha: Data curation, Resources, Writing – review & editing. Julio Huerta‐Espino: Data curation, Resources. Velu Govindan: Resources, Writing – review & editing. Philomin Juliana: Data curation, Resources, Writing – review & editing. Suchismita Mondal: Data curation, Resources. Paulino Pérez‐Rodríguez: Conceptualization, Formal analysis, Writing – original draft. Jose Crossa: Conceptualization, Supervision, Writing – original draft.

## CONFLICT OF INTEREST

The authors declare that there is no conflict of interest.

## Supporting information

Supplemental Figure S1–S3: Distribution of the genomic relationship values between individuals in the prediction year (TST) and individuals in the training (TRN) set consisting of the five previous years, TRN_1‐5_; for TST=2019, 2020, and 2021, respectively. Supplemental Figure S4: Top two principal components of the genomic relationship matrix. Supplemental Figure S5: Mean squared error of prediction of the GBLUP and of the SSI. Supplemental Figures S6‐S8: Genomic relationships versus regression coefficients of the SSI for testing individuals in year TST=2019, 2020, and 2021, respectively. Supplemental Figures S1–S8 are available in the Supplemental Material.

## Data Availability

Phenotypic and genotype data used in this study are available online (https://hdl.handle.net/11529/10548635).
