# Supplementary material for: Sparse kernel models provide optimization of training set design for genomic prediction in multiyear wheat breeding data
Source: Plant Genome. 2022 Aug 31;15(4):e20254. doi: 10.1002/tpg2.20254 (PMC12806882; doi:10.1002/tpg2.20254)
Supplement: Supplementary file 1 [file TPG2-15-e20254-s001.pdf]

Supplemental Material

Sparse kernel models provide optimization of training set design for genome-based prediction in multi-year wheat breeding data

Marco Lopez-Cruz[1], Susanne Dreisigacker[2], Leonardo Crespo-Herrera[2], Alison R Bentley[2], Ravi Singh[2], Jesse Poland[3], Sandesh Shrestha[3], Julio Huerta-Espino[4], Govindan Velu[2], Philomin Juliana[2], Suchismita Mondal[2], Paulino Pérez-Rodríguez[5,*], and Jose Crossa[2,5,*]

[1]Department of Epidemiology and Biostatistics, Michigan State University, East Lansing, MI, USA.
[2]Global Wheat Program, International Maize and Wheat Improvement Center, Mexico
[3]Department of Agronomy, Kansas State University, Manhattan, KS, USA.
[4]Campo Experimental Valle de Mexico, Instituto Nacional de Investigaciones Forestales, Agricolas y Pecuarias (INIFAP), Chapingo, Mexico.
[5]Colegio de Postgraduados, Montecillos, Mexico.

*Corresponding authors: JC (j.crossa@cgiar.org), PPR (perpdgo@gmail.com)

This document includes:

- Pages 1-9
- Supplemental Figures S1-S8

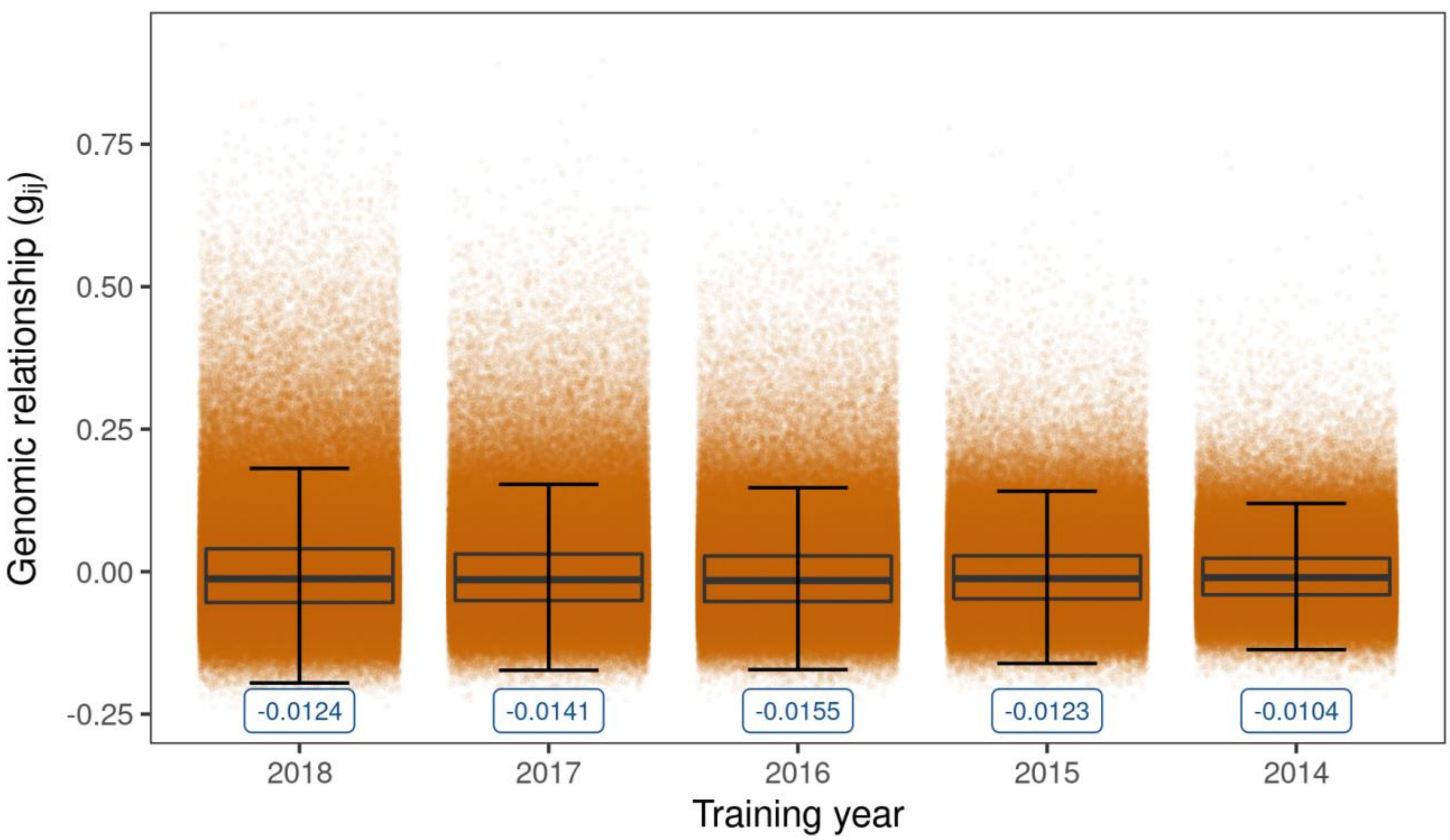

Figure S1. Distribution of the genomic relationship values (g_{ij}) between individuals in the prediction year 2019 ($i = 1, 2, \ldots, 8928$) and individuals in the training (TRN) set consisting of the five previous years ($j = 1, 2, \ldots, 43314$), $TRN_{1\text{-}5}$. Boxplots are separated by each year composing the training set $TRN_{1\text{-}5}$.

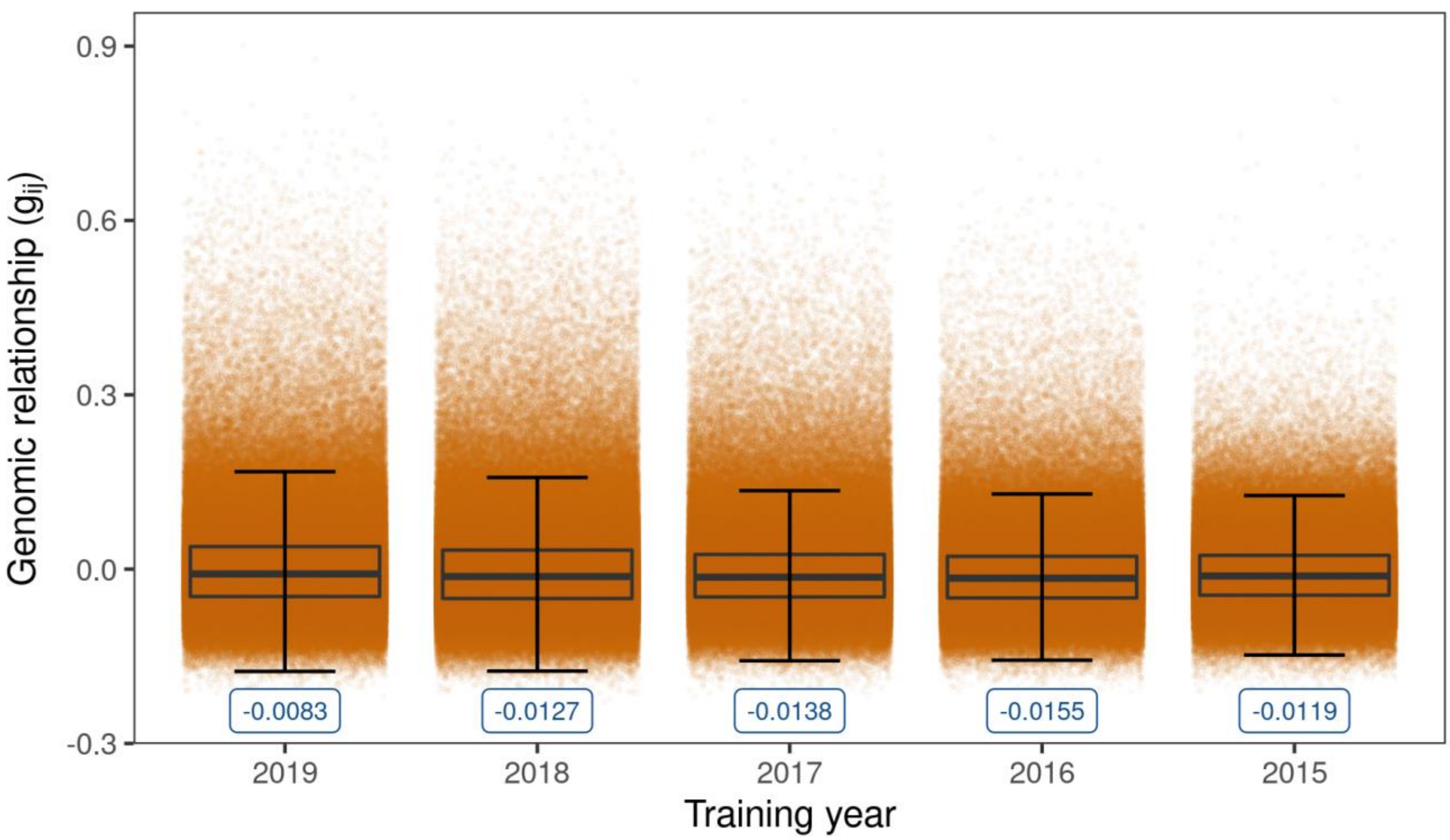

Figure S2. Distribution of the genomic relationship values (g_{ij}) between individuals in the prediction year 2020 ($i = 1, 2, \ldots, 8701$) and individuals in the training (TRN) set consisting of the five previous years ($j = 1, 2, \ldots, 44836$), $TRN_{1\text{-}5}$. Boxplots are separated by each year composing the training set $TRN_{1\text{-}5}$.

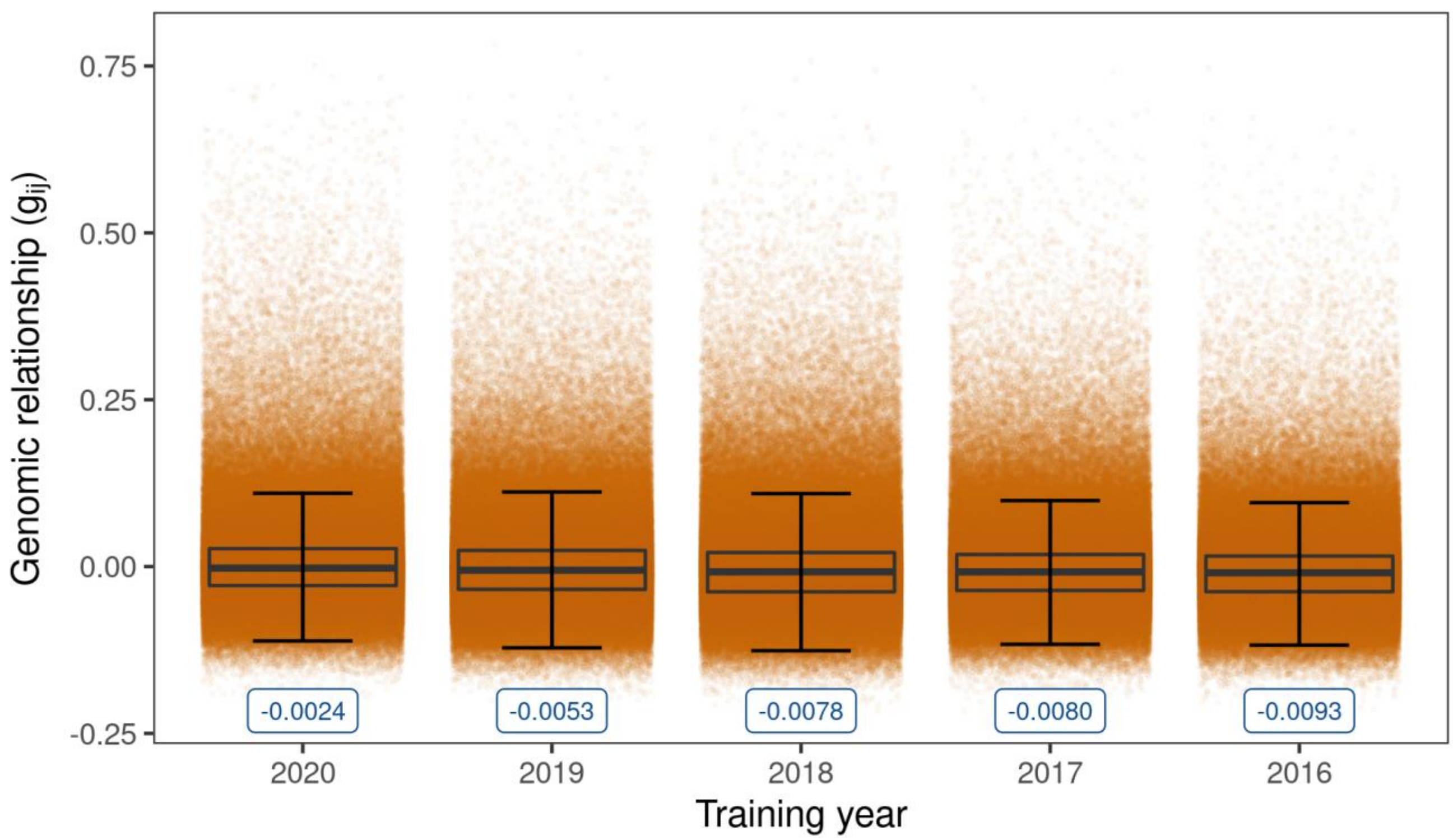

Figure S3. Distribution of the genomic relationship values (g_{ij}) between individuals in the prediction year 2021 ($i = 1, 2, \ldots, 7893$) and individuals in the training (TRN) set consisting of the five previous years ($j = 1, 2, \ldots, 44603$), $TRN_{1\text{-}5}$. Boxplots are separated by each year composing the training set $TRN_{1\text{-}5}$.

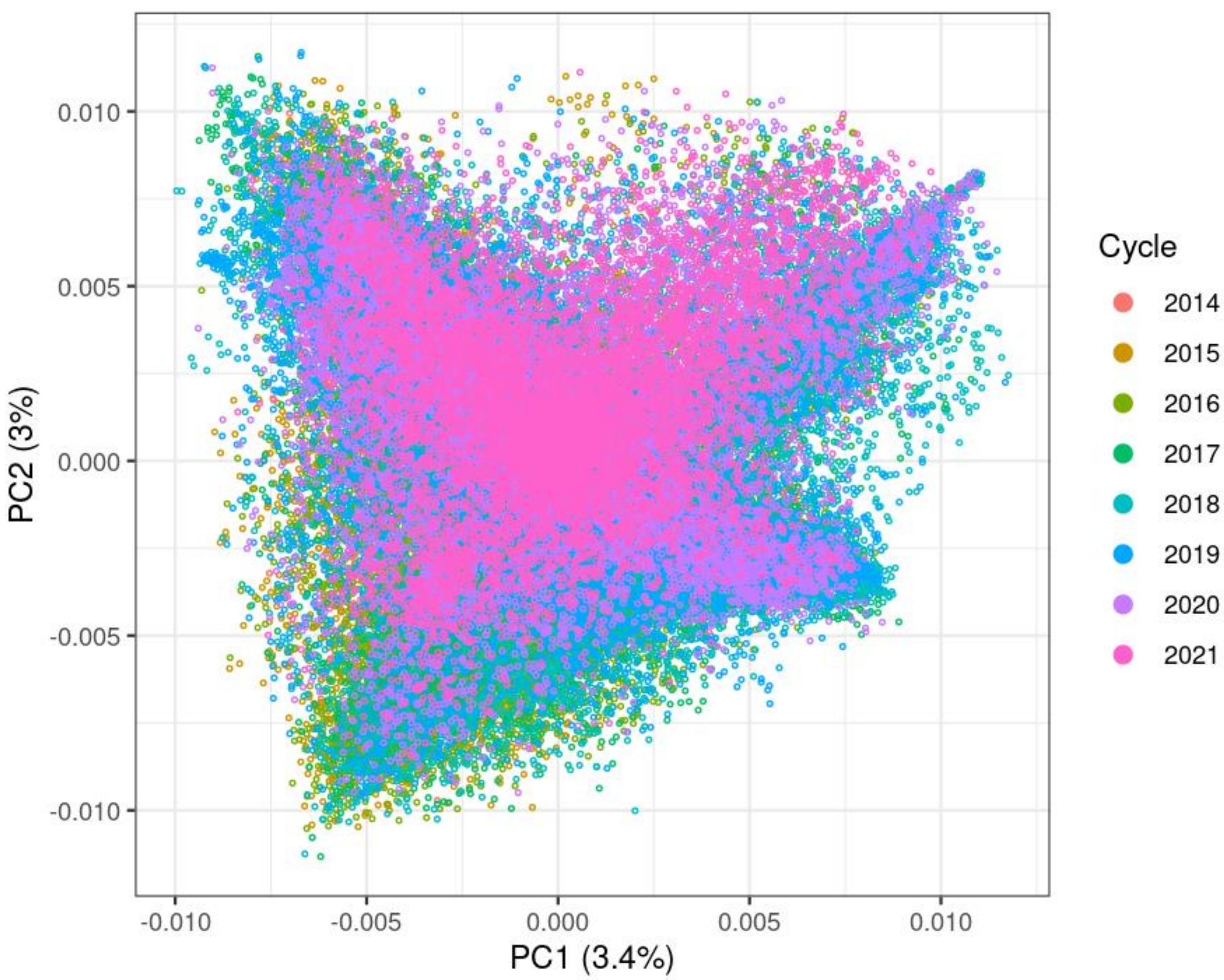

Figure S4. First two principal components of the additive genomic relationship matrix **G** for the $n = 68{,}836$ wheat lines. Each point represents an individual colored by cycle (2014-2021).

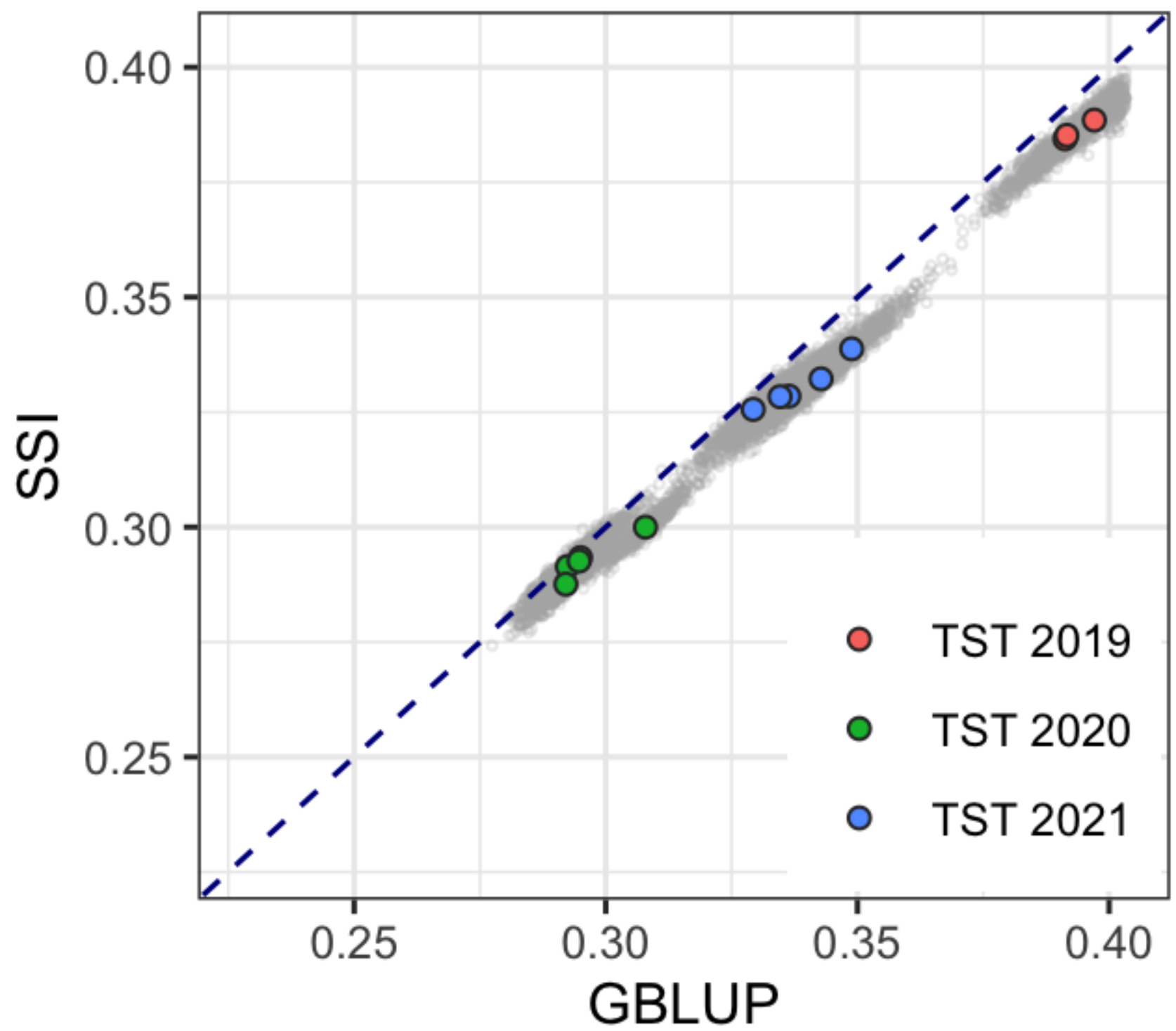

Figure S5. Mean squared error (MSE) of prediction of the GBLUP and of the SSI. Each point represents a prediction case: a prediction (TST) cycle (TST=2019, 2020, or 2021, separated by color) using the five training (TRN) sets (TRN_1, $TRN_{1\text{-}2}$, …, and $TRN_{1\text{-}5}$). Points in gray represent 500 bootstrapped instances for each prediction case. The 45-degree line is shown for comparison, where points above/below the line has a larger/smaller MSE than the other model.

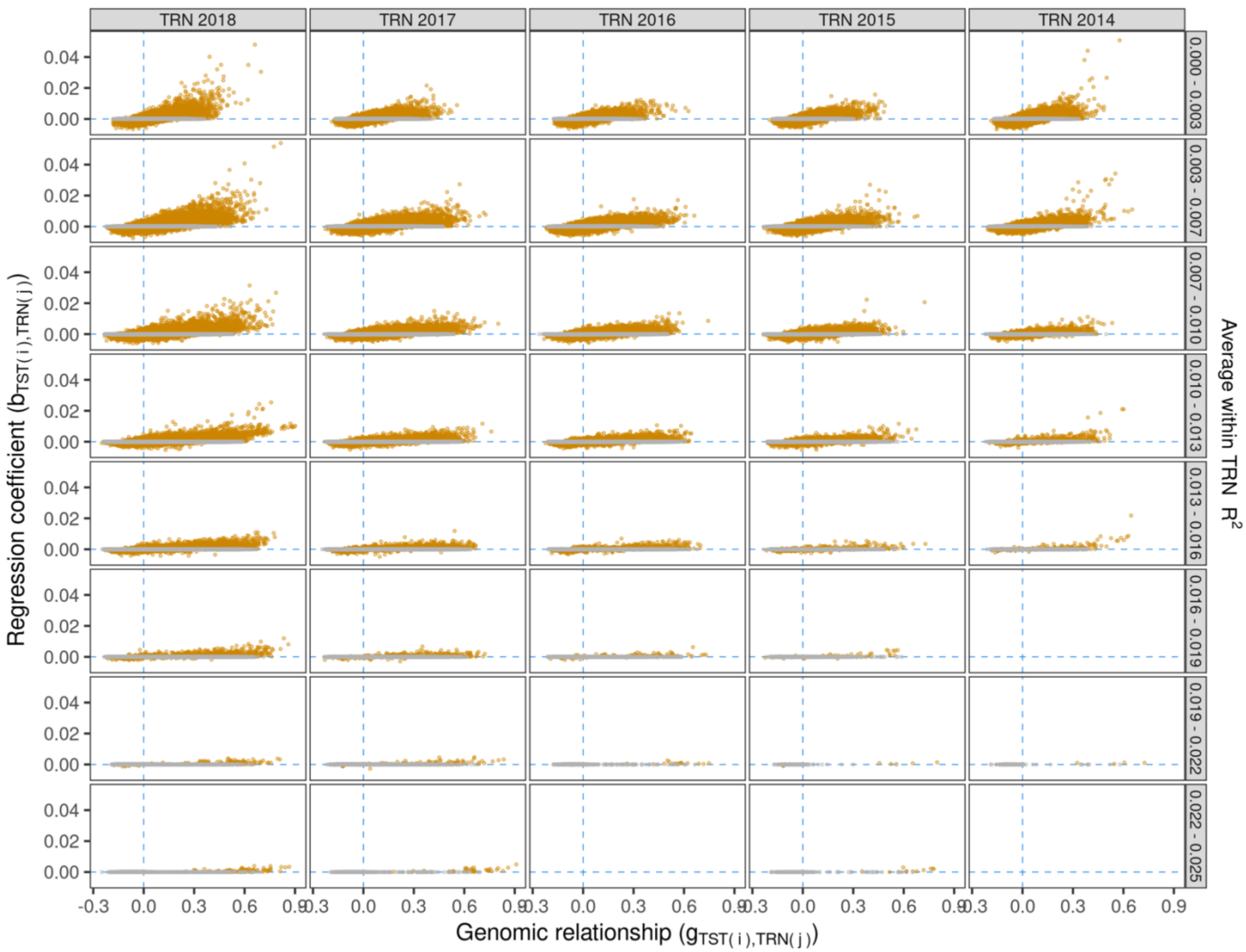

Figure S6. Genomic relationships ($g_{ij} = g_{TST(i)TRN(j)}$) versus regression coefficients ($b_{ij} = b_{TST(i)TRN(j)}$) of the SSI for the testing (TST) individuals in year 2019 ($i = 1, 2, \dots, 8928$). The training (TRN) set consisted of individuals from the five previous years ($j = 1, 2, \dots, 43314$), $TRN_{1\text{-}5}$. TRN individuals are grouped by R^2 level (average R^2 of each TRN individual with the remaining $n_{TRN} - 1$ individuals). Each panel represents a combination of one year composing the $TRN_{1\text{-}5}$ and R^2 level. Points in orange/grey represent training subjects whose coefficient b_{ij} was non-zero/zero.

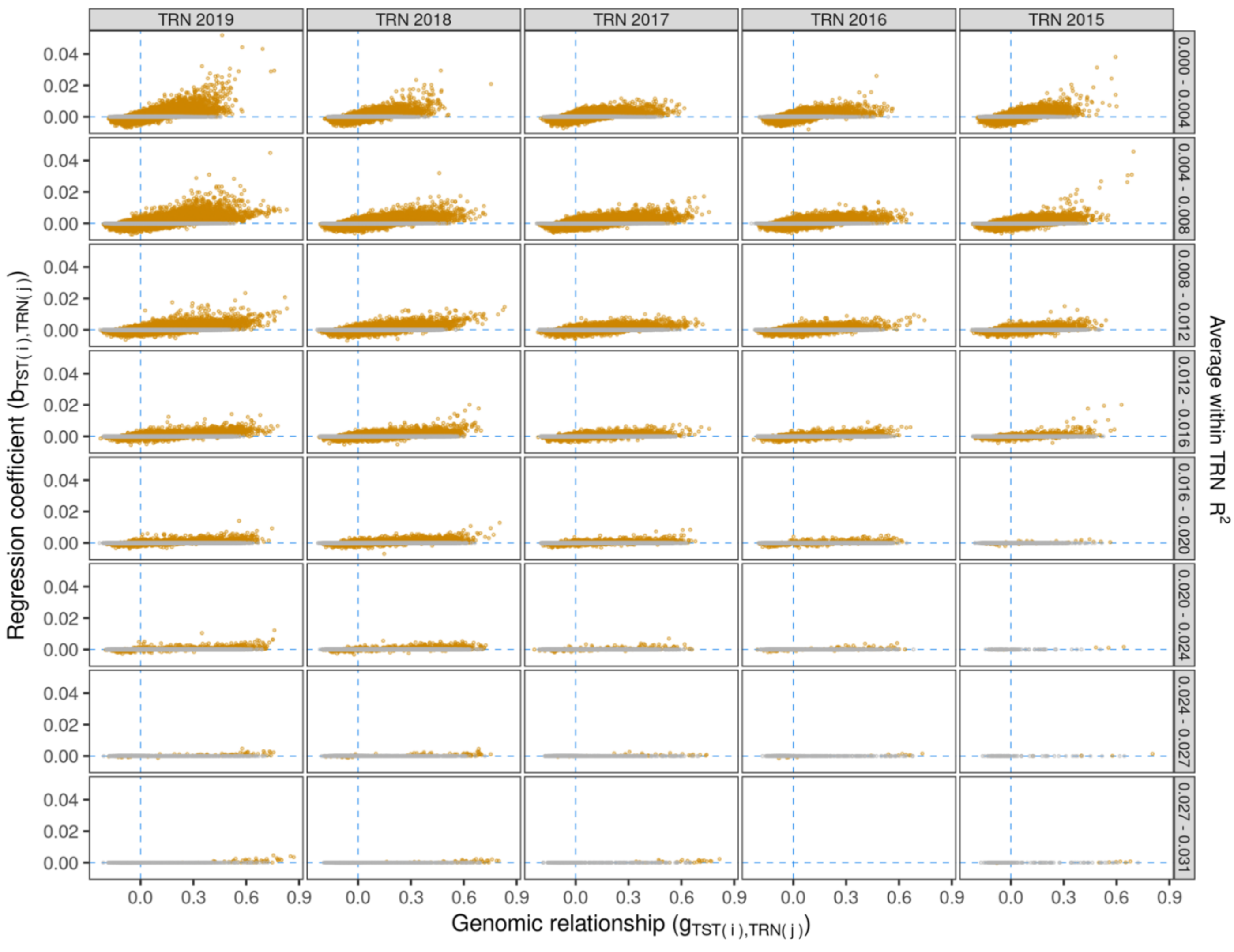

Figure S7. Genomic relationships ($g_{ij} = g_{TST(i)TRN(j)}$) versus regression coefficients ($b_{ij} = b_{TST(i)TRN(j)}$) of the SSI for the testing (TST) individuals in year 2020 ($i = 1, 2, \dots, 8701$). The training (TRN) set consisted of individuals from the five previous years ($j = 1, 2, \dots, 44836$), $TRN_{1\text{-}5}$. TRN individuals are grouped by R^2 level (average R^2 of each TRN individual with the remaining $n_{TRN} - 1$ individuals). Each panel represents a combination of one year composing the $TRN_{1\text{-}5}$ and R^2 level. Points in orange/grey represent training subjects whose coefficient b_{ij} was non-zero/zero.

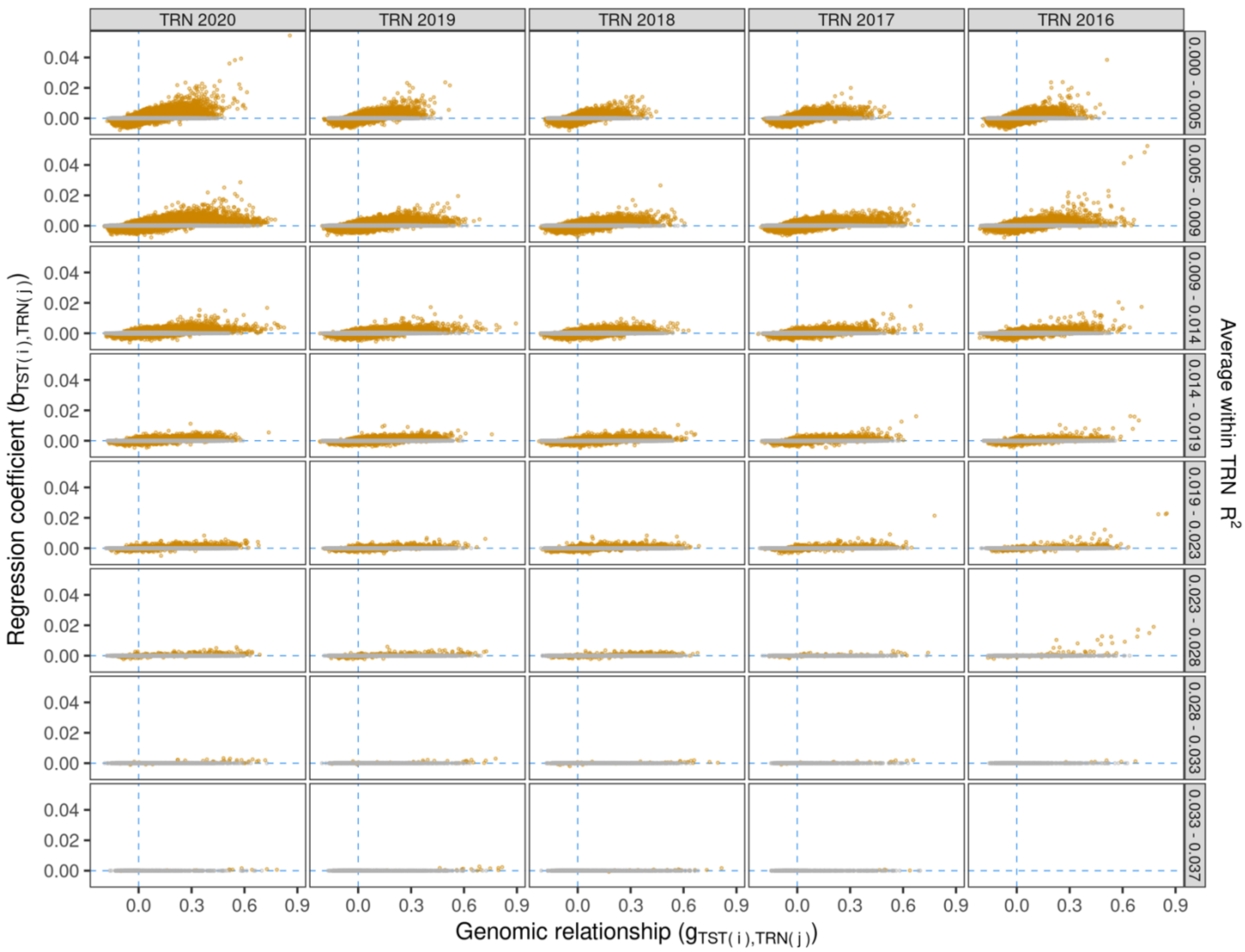

Figure S8. Genomic relationships ($g_{ij} = g_{TST(i)TRN(j)}$) versus regression coefficients ($b_{ij} = b_{TST(i)TRN(j)}$) of the SSI for the testing (TST) individuals in year 2021 ($i = 1, 2, \dots, 7893$). The training (TRN) set consisted of individuals from the five previous years ($j = 1, 2, \dots, 44603$), $TRN_{1\text{-}5}$. TRN individuals are grouped by R^2 level (average R^2 of each TRN individual with the remaining $n_{TRN} - 1$ individuals). Each panel represents a combination of one year composing the $TRN_{1\text{-}5}$ and R^2 level. Points in orange/grey represent training subjects whose coefficient b_{ij} was non-zero/zero.
